# Adrenocortical Carcinoma: A Case of Missed Diagnosis

**DOI:** 10.7759/cureus.14235

**Published:** 2021-04-01

**Authors:** Yusef Hazimeh, Carlie Sigel, Carsello Carie, Mathew Leinung, Zaynab Khalaf

**Affiliations:** 1 Endocrinology, Faculty of Medical Sciences, Lebanese University, Beirut, LBN; 2 Pathology, Memorial Sloan Kettering Cancer Center, New York, USA; 3 Internal Medicine, Albany Medical College, Albany, USA; 4 Endocrinology, Diabetes and Metabolism, Faculty of Medical Sciences, Lebanese University, Beirut, LBN

**Keywords:** adrenal incidentaloma, adrenal cell carcinoma, adrenocortical carcinoma

## Abstract

Incidentalomas are commonly encountered adrenal lesions. However, adrenocortical carcinoma (ACC) represents a rare etiology of adrenal incidentalomas (AI). The diagnosis of AI is generally based on laboratory data and imaging results, Fine needle aspiration (FNA) is not usually indicated in the workup of incidentaloma. In this report, we present a case of AI in which two FNA procedures failed to make the correct diagnosis of ACC.

## Introduction

Adrenal incidentaloma (AI) is a common endocrine condition, and it is defined as an adrenal mass of greater than 1 cm in diameter detected incidentally when performing radiological studies to evaluate a non-adrenal condition or disease. A multidisciplinary workup with a careful laboratory and radiological analysis is required to make a correct diagnosis of the condition and ensure its effective management [1,2]. While AIs are non-functional and benign in most cases, they can be malignant or secreting in approximately 20% of cases [1-3]. Secreting forms of AI include Cushing’s syndrome, pheochromocytoma, and Conn’s disease; they vary significantly in terms of clinical signs and symptoms, with presentations ranging from mild to severe symptoms [1-3]. A personalized approach is required for each case of AI, and it should be based on imaging analysis, endocrine workup, and clinical symptoms and signs. This can help to differentiate among the various etiologies of AI.

Adrenocortical carcinoma (ACC) is a serious medical condition and is found in around 2% of all cases of AI [1]. Functional AI lesions are more common, and they account for around 15% of all cases of AI [1-3]. Studies have shown an increase in the prevalence of AIs with advancing age, with peak incidences occurring between the fifth and seventh decades of life [1]. They are rarely found in patients younger than 30 years of age, and hence such cases, if encountered, should be investigated promptly due to the potential risk of ACC or functional lesions [1,2].

ACC is a rare and aggressive malignant tumor, and it is associated with two different peaks of incidence: before the age of five and between 40-60 years of age; however, it can also occur in people of any age [2]. Most cases of ACC are sporadic but can be associated with other tumor syndromes, such as multiple endocrine neoplasia type 1 (MEN1) gene, Beckwith-Wiedemann syndrome (abnormalities in 11p15I gene), and Li-Fraumeni syndrome (TP53 gene) [1-3].

In this report, we present the case of a patient with AI in whom two fine needle aspiration (FNA) procedures failed to make the correct diagnosis of ACC.

## Case presentation

**Figure 1:**
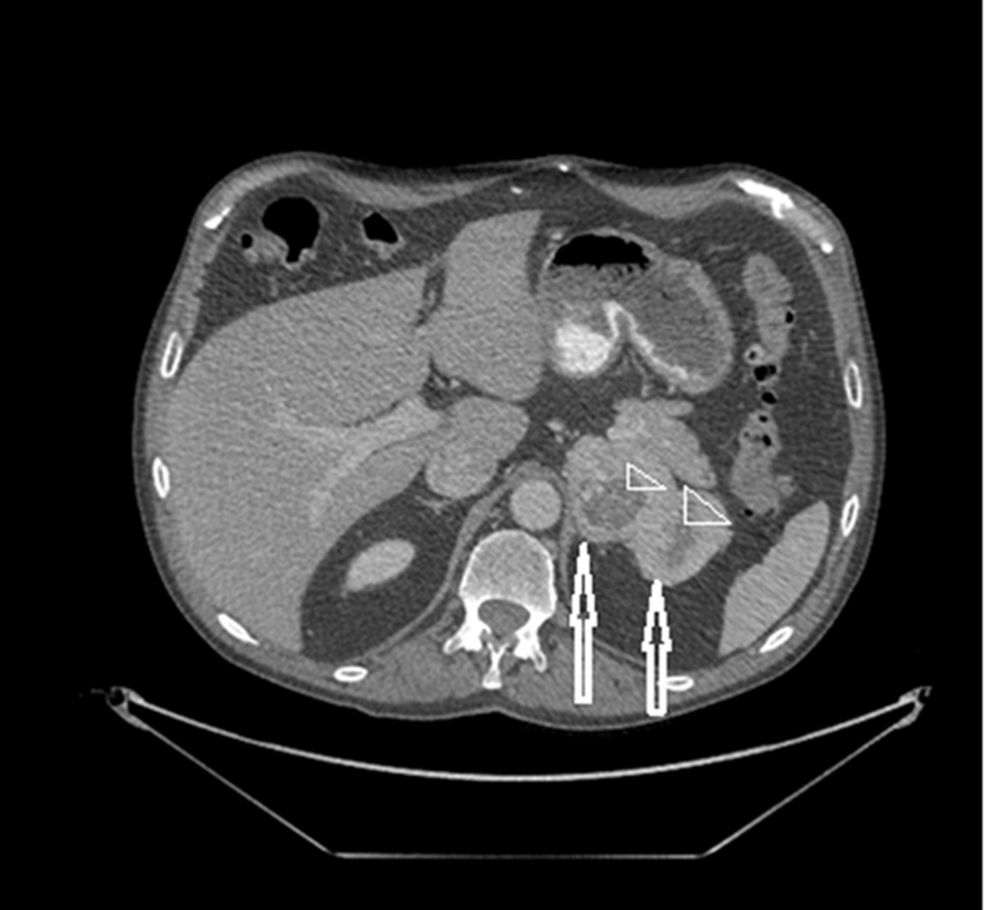
CT scan of the abdomen The image shows a bi-lobed mass (two arrows) in the left adrenal gland measuring 9 x 5.5 cm. Centrally, there are signs of low density indicating necrosis (two arrowheads) CT: computed tomography

A 61-year-old Caucasian male presented to the hospital with a complaint of sudden shortness of breath. CT scan of his lungs was diagnostic for pulmonary emboli and a small lung nodule of 0.5 cm, and incidentally revealed a left adrenal mass of 9 cm. The patient was treated with anticoagulation for pulmonary emboli. He did not show any symptoms of hyper or hypoadrenalism, but serum metanephrine and cortisol were done routinely to determine the secretory status of the adrenal adenoma, and both of them were within normal limits. Biopsy of the left adrenal nodule was ordered by a general practitioner and was performed one month after the discharge, and it was found to be non-diagnostic. However, a repeat biopsy done three weeks after the first one revealed adrenal cortical tissue, raising concerns related to adrenal hyperplasia, adenoma, and unrepresentative biopsy material. The patient was referred to endocrinology three months later. Evaluation by the endocrinologist did not reveal any history of weight gain, weakness, or insomnia. The patient had not observed any change in his libido or hair distribution. He had a history of hypertension for more than 10 years, which was being controlled with two medications. Physical exam showed normal vital signs, no stigmata of Cushing’s syndrome, and normal body mass index (BMI). Urine analysis for catecholamines and cortisol were within normal limits, and so were serum aldosterone-to-renin activity, electrolytes, and dehydroepiandrosterone sulfate (DHEA-S) levels. A repeat CT scan of the abdomen done four months after the first one revealed a bi-lobed heterogeneous mass in the left adrenal gland measuring 9 x 5.5 cm with a central low density likely indicating necrosis (Figure 1).

The mass was suspicious for ACC. A CT scan of the patient's lungs revealed enlargement of the lung nodule to 0.8 cm. He subsequently underwent FNA of the pulmonary nodule.

**Figure 2:**
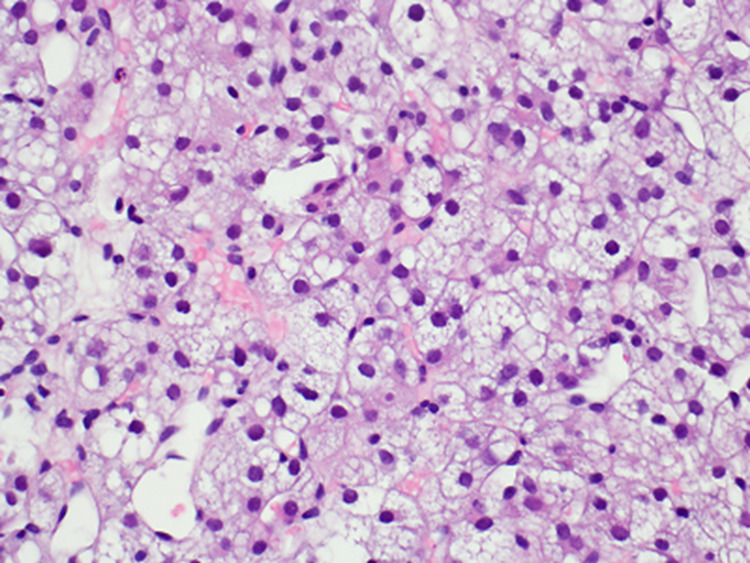
Core biopsy findings of the adrenal gland The biopsy shows adrenal cortical tissue that may be consistent with a neoplasm if corresponding to a mass lesion. No features correlating with aggressive behavior were identified (hematoxylin and eosin stain, 40x magnification)

The adrenal core biopsy and lung nodule fine needle aspirate were sent to a pathologist with expertise in adrenal pathology. Histologic findings from the adrenal gland core biopsy showed cells resembling the adrenal cortex (Figure 2). The nuclei were uniformly low grade and were mildly enlarged, lacked prominent nucleoli, and exhibited fine chromatin.

**Figure 3:**
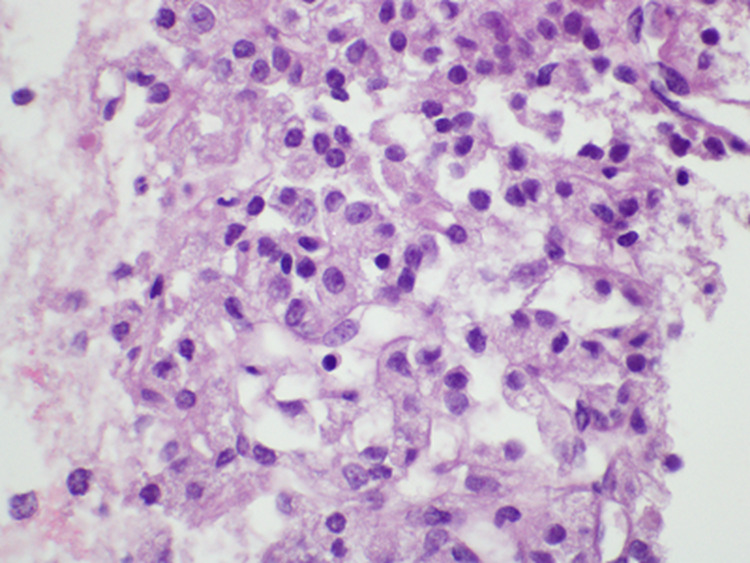
The cell block from the fine needle aspiration of a left lung nodule The cell block demonstrates bland lipid-rich cells similar to the core biopsy findings, indicating metastasis from the adrenal cortical tumor (hematoxylin and eosin stain, 60x magnification)

The majority of cells were lipid-rich and the predominant architectural pattern of growth was nested. The FNA biopsy of a left lung nodule demonstrated similarly bland lipid-rich cells with a capillary-rich network (Figure 3).

The morphologic pattern was very similar to the adrenal biopsy and was consistent with metastatic ACC. The patient subsequently approached a specialized cancer center for treatment. He received mitotane and became adrenally insufficient, and he was started on hydrocortisone.

## Discussion

AIs are a common medical condition, with a reported incidence of about 4% among the general population [4]. The prevalence of AI in older populations may be as high as 10% [5]. However, ACC is very rare, with an incidence rate of approximately one to two cases per million population per year [6].

Clinical syndromes of hormone excess can be present in 45-68% of patients with ACC [7,8]. Although ACC is considered to be an inefficient steroid producer, up to 80% of patients may have some type of adrenal hormonal overproduction issue [8]. Thus, it was easy to miss the diagnosis of ACC in this patient based on the clinical picture and hormonal workup alone. However, signs of malignancy were observed on his CT scan, based on the size and appearance of the adrenal gland. In a series of 1,000 patients with incidentalomas, a size of 4 cm or more was found to have a sensitivity of 93% in differentiating between ACC and benign lesions [9]. Current guidelines from the American Association of Clinical Endocrinologists (AACE) and the American Association of Endocrine Surgeons (AAES) recommend adrenalectomy for all incidentalomas of 4 cm or more [10]. Our patient should have been referred for adrenalectomy instead of biopsy. The bi-lobed feature, heterogeneity, and the presence of central necrosis on the CT scan are commonly seen in ACC with central necrosis reported in up to 80% of the cases [11].

In our case, the initial finding of a 0.5-cm lung nodule was not suspicious for malignancy and the attention was diverted towards treating the patient's pulmonary embolus. However, given the presence of a large adrenal mass, the presence of ACC with lung metastasis should have been considered. In a study of 416 cases of ACC, 29% (122 patients) presented with distant metastasis; the primary extra-adrenal organ involved was the lungs (65%, 79/122 patients) [12].

## Conclusions

Even though ACC is a rare condition, it should be suspected in patients with AIs that show certain characteristics on imaging. FNA is not recommended as part of the evaluation and can be misleading. Current guidelines recommend the removal of lesions of >4 cm.
